# Supplementary material for: Users’ needs for a digital smoking cessation application and how to address them: A mixed-methods study
Source: PeerJ. 2022 Aug 19;10:e13824. doi: 10.7717/peerj.13824 (PMC9394512; doi:10.7717/peerj.13824)
Supplement: Supplemental Information 1 — Titles and formulations for the 24 preparatory activities that were used in the study, 12 each for smoking cessation and physical activity increase. 4 activities had another activity as prerequisite. [file peerj-10-13824-s001.pdf]

Table S1: **Preparatory activities**. Titles and formulations for the 24 preparatory activities that were used in the study, 12 each for smoking cessation and physical activity increase. 4 activities had another activity as prerequisite.

	**Title and Formulation**	**Prerequisite**
Smoking cessation		
1	**Desired future self (writing)**. Having high aspiration to quit smoking may aid quitting successfully. Thus, before the next session, I advise you to think about the person that you would like to be once you have successfully quit smoking. For example, you might want to be a ”grandfather who can play football with his grandchildren” or a ”nurse who can walk up the stairs to the fourth floor without getting out of breath.” Write down everything that comes to your mind.	
2	**Reasons for quitting smoking**. Having high aspiration to quit smoking may aid quitting successfully. So, before the next session, I advise you to identify and write down reasons why you want to stop smoking. After writing them down, think about which reasons are most important to you and order them accordingly.	
3	**Feared future self (writing)**. Having high motivation to quit smoking may aid quitting successfully. Thus, before the next session, I advise you to think about who you do NOT want to be in the future but might become if you continue to smoke. For example, you might NOT want to be a ”mother who dies early of coronary heart disease like her mother did” or a ”husband who is frowned upon by his wife” or a ”man who is dependent on a substance.” Write down everything that comes to your mind.	
4	**Feared future self (picture)**. Having high motivation to quit smoking may help to quit successfully. So, before the next session, I advise you to think about who you do NOT want to be in the future but might become if you continue to smoke. For example, you might NOT want to be a ”mother who dies early of coronary heart disease like her mother did” or a ”husband who is frowned upon by his wife” or a ”man who is dependent on a substance.” Then, look for or take a picture that best captures your feared future self. Save or print this picture so that you can see it every day.	
5	**Fighting match visualization**. Focusing on your goal of successfully quitting smoking may help to quit. Thus, before the next session, I advise you to take some time to visualize smoking as a battle. For example, you might see yourself and a cigarette as two boxers in a fighting match. Then imagine yourself winning this battle. Visualize clearly how you win and what it feels like to be the winner. Write down a few words about your winning experience.	
6	**Smoking-relevant routines**. Getting fewer cravings to smoke may make it easier to successfully quit smoking. Therefore, before the next session, I advise you to think about routines in your daily life that often cause you to get cravings to smoke. For example, you might have experienced that if you go to bed very late and thus sleep less, you smoke more the next day. Or maybe you have noticed that if you skip your breakfast, you always smoke on your way to work but NOT otherwise. How could you change these routines to reduce or even avoid those cravings? Write down everything that comes to your mind.	12
7	**High risk situations**. Preparing for situations in which avoiding to smoke is difficult may make it easier to successfully quit smoking. Thus, before the next session, I advise you to think about situations in which you might find it difficult to refrain from smoking. For example, this could be during your lunch break at work, when you meet your best friend, or when you watch TV. How could you deal with these situations so that you do NOT smoke? Write down your plans in a few words.	12

	Title and Formulation	Prerequisite
8	**Coping with cravings**. Planning how to resist urges to smoke may make it easier to successfully quit smoking. Therefore, before the next session, I advise you to think about activities that you could do to keep yourself busy when you feel the urge to smoke so that you do NOT smoke. These urges typically last a few minutes; think about something that you could do in the meantime until the urge has passed. For example, you could water your plants, eat a carrot, do 10 push-ups, or do something for another person in need. Write down everything that comes to your mind.	
9	**Relaxation exercise**. Tensing and relaxing areas of the body can reduce cravings and withdrawal symptoms, because it is very difficult to feel tense or uptight in a relaxed body. Thus, before the next session, I advise you to watch the following 15-minute video to learn progressive muscle relaxation (which is a way of relaxing your body): `https://www.youtube.com/watch?v=ihO02wUzgkc&ab_channel=MarkConnelly`. Even if you have already heard of this technique, it might be a good idea to refresh your memory.	
10	**Personal rule**. Having strong determination to refrain from smoking may help to quit successfully. So, before the next session, I advise you to take some time to create a personal rule that helps you to refrain from smoking. Possible examples include "Not a puff - no matter what," "Say no to smoking, yes to life" or "Smoking is NOT an option." Write down your rule on a piece of paper and repeat it to yourself 3 times. Put the piece of paper with your rule somewhere you can see it every day.	
11	**Education on body repair**. Having a strong desire to refrain from smoking may aid quitting successfully. Thus, before the next session, I advise you to watch the following 12-minute video on how the body starts repairing itself immediately, as soon as a person stops smoking: `https://www.youtube.com/watch?v=ZhTOCOT3P3c&ab_channel=RespiratoryTherapyZone`. What information from the video is most relevant to you? Take a few notes.	
12	**Recording smoking behavior**. Preparing for situations in which you commonly smoke may make it easier to successfully quit smoking. Therefore, I recommend that you record the situations in which you smoke before the next session. Take note of one or two keywords to describe the situation and the number of cigarettes that you smoked. For example, you might note "Lunch break, 2 cigarettes" or "TV, 5 cigarettes." It might be helpful to take these notes on your phone, or you could carry a small piece of paper and pen in your pocket.	
PHYSICAL ACTIVITY INCREASE		
13	**Recording physical activity**. Becoming more physically active (e.g. exercise, take walks, sit less) may make it easier to successfully quit smoking. One important step for becoming more physically active is to know one's current level. This allows to later set a precise goal and hence to feel more motivated. So, I recommend that you record your current behavior with regards to physical activity before the next session. Try to keep track of how much time you spend 1) sitting, 2) working out and 3) being moderately active (e.g. taking a walk, biking to the grocery store). For this, it might be helpful to keep a piece of paper and pen on your kitchen table, or maybe you have a smart watch that can record these types of behavior for you.	
14	**Barriers**. Becoming more physically active (e.g. exercise, take walks, sit less) may help you to successfully quit smoking. One important step for becoming more physically active is to remove possible obstacles. Thus, before the next session, I advise you to think about things that make it difficult for you to be physically active. For example, this could be that you do NOT have a raincoat to bike to the grocery store when it is raining, that you do NOT want to work out alone, or that you are at work all day and too exhausted by the time that you come home. What are possible solutions to your barriers? For instance, you could buy a raincoat, join a running group, or take a walk during your lunch break at work. Write down everything that comes to your mind.	

	Title and Formulation	Prerequisite
15	**Reasons for becoming more physically active**. Quitting smoking may be easier if you become more physically active (e.g. exercise, take walks, sit less). One crucial step for this is to have a strong desire to become more physically active. Therefore, before the next session, I advise you to identify and write down reasons why you want to become more physically active. After writing them down, think about which reasons are most important to you and order them accordingly.	
16	**Recommended physical activity**. Quitting smoking may be easier if you become more physically active (e.g. exercise, take walks, sit less). One important step for becoming more physically active is to set a specific goal and thus to feel more aspiration. Therefore, before the next session, I advise you to watch the following 2-minute video on how much and which type of physical activity is recommended: `https://www.youtube.com/watch?v=AAPhWbG_zLs&ab_channel=TREKGroup`. Then, compare your physical activity behavior to the recommended amounts for the different types of physical activity. Write down which recommended amounts you meet or exceed, and which ones you do NOT meet.	13
17	**Desired future self (writing)**. Quitting smoking may be easier if you become more physically active (e.g. exercise, take walks, sit less). One important step for this is to have high ambition to become more physically active. Thus, before the next session, I advise you to think about the person that you would like to be once you have become more physically active. For example, you might want to be a ”grandfather who can play football with his grandchildren” or a ”nurse who can walk up the stairs to the fourth floor without getting out of breath.” Write down everything that comes to your mind.	
18	**Feared future self (writing)**. It may be easier to successfully quit smoking if you become more physically active (e.g. exercise, take walks, sit less). One crucial step for this is to have high determination to become more physically active. Therefore, before the next session, I advise you to think about who you do NOT want to be in the future but might become if you fail to become more physically active. For example, you might NOT want to be a ”mother who dies early of coronary heart disease like her father did” or a ”daughter who is frowned upon by her mother” or a ”man who is dependent on his wife in his everyday life.” Write down everything that comes to your mind.	
19	**Feared future self (picture)**. It may be easier to successfully quit smoking if you become more physically active (e.g. exercise, take walks, sit less). One crucial step for this is to have high determination to become more physically active. Therefore, before the next session, I advise you to think about who you do NOT want to be in the future but might become if you fail to become more physically active. For example, you might NOT want to be a ”mother who dies early of coronary heart disease like her father did” or a ”daughter who is frowned upon by her mother” or a ”man who is dependent on his wife in his everyday life.” Then, look for or take a picture that best captures your feared future self. Save or print this picture so that you can see it every day.	
20	**Fighting match visualization**. Becoming more physically active (e.g. exercise, take walks, sit less) may help you to successfully quit smoking. One important step for this is to focus on the goal of becoming more physically active. Thus, before the next session, I advise you to take some time to visualize becoming more physically active as a battle. For example, you might see yourself and non-active version of yourself as two boxers in a fighting match. Then imagine yourself winning this battle. Visualize clearly how you win and what it feels like to be the winner. Write down a few words about your winning experience.	

	Title and Formulation	Prerequisite
21	**Desired future self (picture)**. Quitting smoking may be easier if you become more physically active (e.g. exercise, take walks, sit less). One crucial step for this is to have high motivation to become more physically active. Thus, before the next session, I advise you to think about the person that you would like to be once you have become more physically active. For example, you might want to be a ”grandfather who can play football with his grandchildren” or a ”nurse who can walk up the stairs to the fourth floor without getting out of breath.” Then look for or take a picture that best captures your desired future self. Save or print this picture so that you can see it every day.	
22	**Plan for becoming more physically active**. Becoming more physically active (e.g. exercise, take walks, sit less) may help you to successfully quit smoking. One crucial part for this is to create a plan for becoming more physically active. Therefore, before the next session, I advise you to think about what you could do to become more physically active. For example, you could get up from your desk after every 30 minutes of sitting, bike to the grocery store, do 10 squats every morning, or join a running group. Write down everything that comes to your mind. Which plan do you want to focus on? Highlight this plan.	13
23	**Impact of physical activity on cravings**. Becoming more physically active (e.g. exercise, take walks, spend less time sitting) may help you to successfully quit smoking. One crucial step for this is to have high aspiration to become more physically active. So, before the next session, I advise you to watch the following 5-minute video about the possible positive impact of physical activity on dealing with cravings to smoke: `https://www.youtube.com/watch?v=StM10jzbt1k&ab_channel=TreeHouseRecovery`. What do you think about the information in the video? Write down your thoughts in a few words.	
24	**Personal rule**. Being more physically active (e.g. exercise, take walks, spend less time sitting) may aid you to stop smoking. One important aspect for this is to have strong resolve to become more physically active. So, before the next session, I advise you to take some time to create a personal rule that helps you to become more physically active. Possible examples include ”10 squats - no matter what,” ”Say no to sitting, yes to life” or ”Driving to the grocery store is NOT an option.” Write down your rule on a piece of paper and repeat it to yourself 3 times. Put the piece of paper with your rule somewhere you can see it every day.	
