# Supplementary material for: Users’ needs for a digital smoking cessation application and how to address them: A mixed-methods study
Source: PeerJ. 2022 Aug 19;10:e13824. doi: 10.7717/peerj.13824 (PMC9394512; doi:10.7717/peerj.13824)
Supplement: Supplemental Information 2 — Some scenarios about similar interactions are grouped together to facilitate their analysis. [file peerj-10-13824-s002.pdf]

Table S2: **Interaction scenarios.** Some scenarios about similar interactions are grouped together to facilitate their analysis.

Scenario	Topic	Scenario Group
INTERACTION WITH VIRTUAL COACH		
1	Follow physical activity program while quitting smoking	Follow PA program
2	Plan for smoking HRSs in the mornings	Regular HRS planning/reflection
3	Plan for physical activity HRSs on Sundays	Regular HRS planning/reflection
4	Help button for smoking HRSs	Help button for HRS
5	Help button for physical activity HRSs	Help button for HRS
6	Reflect on smoking HRSs in the evenings	Regular HRS planning/reflection
7	Reflect on physical activity HRSs on Sundays	Regular HRS planning/reflection
8	Discuss repeated failure of reaching physical activity goals	Discuss repeated failure PA
9	Receive motivational messages	Receive motivational messages
INTERACTION WITH SOCIAL ENVIRONMENT		
10	Tell SE about quit attempt	Involve SE
11	Discuss with an SO how they can support the quit attempt	Involve SE
INTERACTION WITH GENERAL PRACTITIONER		
12	Consult GP at start of quit attempt	Involve GP
13	Consult GP in case of smoking relapse	Involve GP

Abbreviations: PA, Physical activity; HRS, High risk situation; SE, Social environment; SO, Significant other; GP, General practitioner.
