# Supplementary material for: Users’ needs for a digital smoking cessation application and how to address them: A mixed-methods study
Source: PeerJ. 2022 Aug 19;10:e13824. doi: 10.7717/peerj.13824 (PMC9394512; doi:10.7717/peerj.13824)
Supplement: Supplemental Information 3 — We provide links to both the male and the female version. [file peerj-10-13824-s003.pdf]

Table S3: **Links to the videos of the interaction scenarios**. We provide links to both the male and the female version.

Scenario	Topic	Link to Video
INTERACTION WITH VIRTUAL COACH		
1	Follow physical activity program while quitting smoking	Female: https://youtu.be/_0lmuAuJbfU, Male: https://youtu.be/gHZZKX6dQMo
2	Plan for smoking HRSs in the mornings	Female: https://youtu.be/DyZPA781nn4, Male: https://youtu.be/zpWy28vtSzM
3	Plan for physical activity HRSs on Sundays	Female: https://youtu.be/JFzl7RAXD0U, Male: https://youtu.be/cA78pzMoFuo
4	Help button for smoking HRSs	Female: https://youtu.be/aLrCZyFvslo, Male: https://youtu.be/sLBF2BuMv80
5	Help button for physical activity HRSs	Female: https://youtu.be/5j1a6Nlhs8s, Male: https://youtu.be/HZLtG4LfvIs
6	Reflect on smoking HRSs in the evenings	Female: https://youtu.be/dkUkzneVDyM, Male: https://youtu.be/Raq7Qyvh45A
7	Reflect on physical activity HRSs on Sundays	Female: https://youtu.be/nczb97qrg0g, Male: https://youtu.be/FXC7oZLLzvQ
8	Discuss repeated failure of reaching physical activity goals	Female: https://youtu.be/UHdslaEBlGQ, Male: https://youtu.be/STO8tEGpWXI
9	Receive motivational messages	Female: https://youtu.be/eV6TLfa-hIs. Male: https://youtu.be/IGKRjhK40Cs
INTERACTION WITH SOCIAL ENVIRONMENT		
10	Tell SE about quit attempt	Female: https://youtu.be/SozlTeF0EIY, Male: https://youtu.be/wDxMd-vwJgM
11	Discuss with an SO how they can support the quit attempt	Female: https://youtu.be/UPxHprogZc0, Male: https://youtu.be/ZbopPvhQJWw
INTERACTION WITH GENERAL PRACTITIONER		
12	Consult GP at start of quit attempt	Female: https://youtu.be/lDTeHgBA71w, Male: https://youtu.be/2WJQDn251DM
13	Consult GP in case of smoking relapse	Female: https://youtu.be/Rl-xdeRexUw, Male: https://youtu.be/w5W1DGHKTn4

Abbreviations: HRS, High risk situation; SE, Social environment; SO, Significant other; GP, General practitioner.
