# Supplementary material for: Users’ needs for a digital smoking cessation application and how to address them: A mixed-methods study
Source: PeerJ. 2022 Aug 19;10:e13824. doi: 10.7717/peerj.13824 (PMC9394512; doi:10.7717/peerj.13824)
Supplement: Supplemental Information 4 [file peerj-10-13824-s004.pdf]

Table S4: **Details on how we measured the activity feedback as well as barriers and motivators for doing the preparatory activities.**

Measure	Explanation	Specification	Source
Activity Feedback			
Activity effort	Effort spent on activity from previous session	Scale from 0 ("Nothing") to 10 ("Extremely strong").	Adapted based on Hutchinson and Tenenbaum (2006).
Activity experience	Experience with activity from previous session	Free-text response to the question "How did you approach, do, or experience your assigned activity?" If the effort spent on an activity was lower than four, the question was supplemented by the sentence "If you have no feedback on the activity, just type 'None' in the text field." In the post-questionnaire, this additional sentence was provided independent of the activity effort.	
Activity experience modification	Changes and/or additions for the activity experience	After a user had provided an answer to the activity experience question, the virtual coach played the answer back to the user and gave them the option to make changes or additions. If a user indicated that they would like to make a change or addition, they were asked to provide another free-text response.	
Barriers and Motivators			
Barriers	Barriers for doing the activities	Free-text response to the question "What were barriers for you to do your assigned activities? Barriers are factors that may have restricted, impeded, or blocked your completion of your activities. Please enter "None" if there was nothing and nobody that restricted, impeded, or blocked your completion of your activities."	Definition of barrier in the APA Dictionary of Psychology (American Psychological Association, 2021).
Motivators	Motivators for doing the activities	Free-text response to the question "What aspects motivated you to do your assigned activities? Please enter "None" if there was nothing and nobody that motivated you."	

American Psychological Association. 2021. Apa dictionary of psychology - barrier. *Available at*: `https://dictionary.apa.org/barrier`.

Hutchinson JC, Tenenbaum G. 2006. Perceived effort—can it be considered gestalt? *Psychology of Sport and Exercise* **7(5)**:463–476 DOI 10.1016/j.psychsport.2006.01.007.
