# Supplementary material for: Users’ needs for a digital smoking cessation application and how to address them: A mixed-methods study
Source: PeerJ. 2022 Aug 19;10:e13824. doi: 10.7717/peerj.13824 (PMC9394512; doi:10.7717/peerj.13824)
Supplement: Supplemental Information 5 [file peerj-10-13824-s005.pdf]

Table S5: **Question and scale endpoints for each interaction scenario.**

| Scenario | Topic | Question | Scale Endpoints |
|---|---|---|---|
| Interaction with virtual coach | | | |
| 1 | Follow physical activity program while quitting smoking | If this were you, would you follow this advice and also follow the program for becoming more physically active? | -5 ("Definitely no"), 5 ("Definitely yes") |
| 2 | Plan for smoking HRSs in the mornings | If this were you, would you make such a plan with your virtual coach in the morning? | -5 ("Definitely no"), 5 ("Definitely yes") |
| 3 | Plan for physical activity HRSs on Sundays | If this was you, would you make such a plan with your virtual coach on Sundays? | -5 ("Definitely no"), 5 ("Definitely yes") |
| 4 | Help button for smoking HRSs | If this was you, would you press the help button when you have a craving? | -5 ("Definitely no"), 5 ("Definitely yes") |
| 5 | Help button for physical activity HRSs | If this was you, would you press the help button in this situation? | -5 ("Definitely no"), 5 ("Definitely yes") |
| 6 | Reflect on smoking HRSs in the evenings | If this was you, would you follow this advice and consult your virtual coach in the evening? | -5 ("Definitely no"), 5 ("Definitely yes") |
| 7 | Reflect on physical activity HRSs on Sundays | If this was you, would you consult your virtual coach on Sundays? | -5 ("Definitely no"), 5 ("Definitely yes") |
| 8 | Discuss repeated failure of reaching physical activity goals | If this was you, would you take this advice and consult your virtual coach? | -5 ("Definitely no"), 5 ("Definitely yes") |
| 9 | Receive motivational messages | If this were you, what do you think would be the impact of such a motivational message on you doing the activity? | -5 ("Definitely negative"), 5 ("Definitely positive") |
| Interaction with social environment | | | |
| 10 | Tell SE about quit attempt | If this was you, would you follow up on this advice and tell your social environment about your quit attempt? | -5 ("Definitely no"), 5 ("Definitely yes") |
| 11 | Discuss with an SO how they can support the quit attempt | If this were you, would you follow up on this advice and reach out to a significant other for additional support? | -5 ("Definitely no"), 5 ("Definitely yes") |
| Interaction with general practitioner | | | |
| 12 | Consult GP at start of quit attempt | If this was you, would you follow up on this advice and contact your GP? | -5 ("Definitely no"), 5 ("Definitely yes") |
| 13 | Consult GP in case of smoking relapse | If this was you, would you contact your GP if your app advises you to do so? | -5 ("Definitely no"), 5 ("Definitely yes") |

Abbreviations: HRS, High risk situation; SE, Social environment; SO, Significant other; GP, General practitioner.
