# Supplementary material for: Users’ needs for a digital smoking cessation application and how to address them: A mixed-methods study
Source: PeerJ. 2022 Aug 19;10:e13824. doi: 10.7717/peerj.13824 (PMC9394512; doi:10.7717/peerj.13824)
Supplement: Supplemental Information 6 — Characteristics of the 671 participants with at least one valid free-text response. [file peerj-10-13824-s006.pdf]

Table S6: **Participant characteristics.** Characteristics of the 671 participants with at least one valid free-text response.

**Characteristic**	**Value**
Age	
- 18 – 30, n (%)	315 (46.94%)
- 31 – 40, n (%)	168 (25.04%)
- 41 – 50, n (%)	100 (14.90%)
- 51 – 60, n (%)	70 (10.43%)
- 61 – 74, n (%)	18 (2.68%)
Gender	
- Female, n (%)	349 (52.01%)
- Male, n (%)	310 (46.20%)
- Other, n (%)	11 (1.64%)
- *No data*, n (%)	1 (0.15%)
Highest completed education level	
- No formal qualifications, n (%)	5 (0.75%)
- Secondary education (e.g. GED/GCSE), n (%)	76 (11.33%)
- High school diploma/A-levels, n (%)	170 (25.34%)
- Technical/community college, n (%)	103 (15.35%)
- Undergraduate degree (BA/BSc/other), n (%)	211 (31.45%)
- Graduate degree (MA/MSc/MPhil/other), n (%)	95 (14.16%)
- Doctorate degree (PhD/other), n (%)	7 (1.04%)
- *No data*, n (%)	4 (0.60%)
Previous quit attempt of at least 24 hours	
- Yes, n (%)	528 (78.69%)
- No, n (%)	143 (21.31%)
Smoking frequency	
- Once a day, n (%)	36 (5.37%)
- 2 – 5 times a day, n (%)	165 (24.59%)
- 6 – 10 times a day, n (%)	213 (31.74%)
- 11 – 19 times a day, n (%)	190 (28.32%)
- More than 20 times a day, n (%)	64 (9.54%)
- *No data*, n (%)	3 (0.45%)
TTM-stage for becoming physically active	
- Precontemplation, n (%)	44 (6.56%)
- Contemplation, n (%)	188 (28.02%)
- Preparation, n (%)	159 (23.70%)
- Action, n (%)	83 (12.37%)
- Maintenance, n (%)	197 (29.36%)
TTM-stage for quitting smoking	
- Contemplation, n (%)	577 (85.99%)
- Preparation, n (%)	94 (14.01%)
Weekly exercise amount	
- Never (0 – 60 minutes per week), n (%)	174 (25.93%)
- Sometimes (60 – 150 minutes per week), n (%)	322 (47.99%)
- Often (more than 150 minutes per week), n (%)	170 (25.34%)
- *No data*, n (%)	5 (0.75%)

Abbreviations: GED, General educational development; GCSE, General certificate of secondary education; BA, Bachelor of Arts; BSc, Bachelor of Science; MA, Master of Arts; MSc, Master of Science; MPhil, Master of Philosophy; PhD, Doctor of Philosophy; TTM, Transtheoretical model.
