# Supplementary material for: Users’ needs for a digital smoking cessation application and how to address them: A mixed-methods study
Source: PeerJ. 2022 Aug 19;10:e13824. doi: 10.7717/peerj.13824 (PMC9394512; doi:10.7717/peerj.13824)
Supplement: Supplemental Information 7 — The coding scheme consists of 4 codes at the highest level, 15 codes at the second level, and 86 codes at the third level. We show the reliability for each coding level. [file peerj-10-13824-s007.pdf]

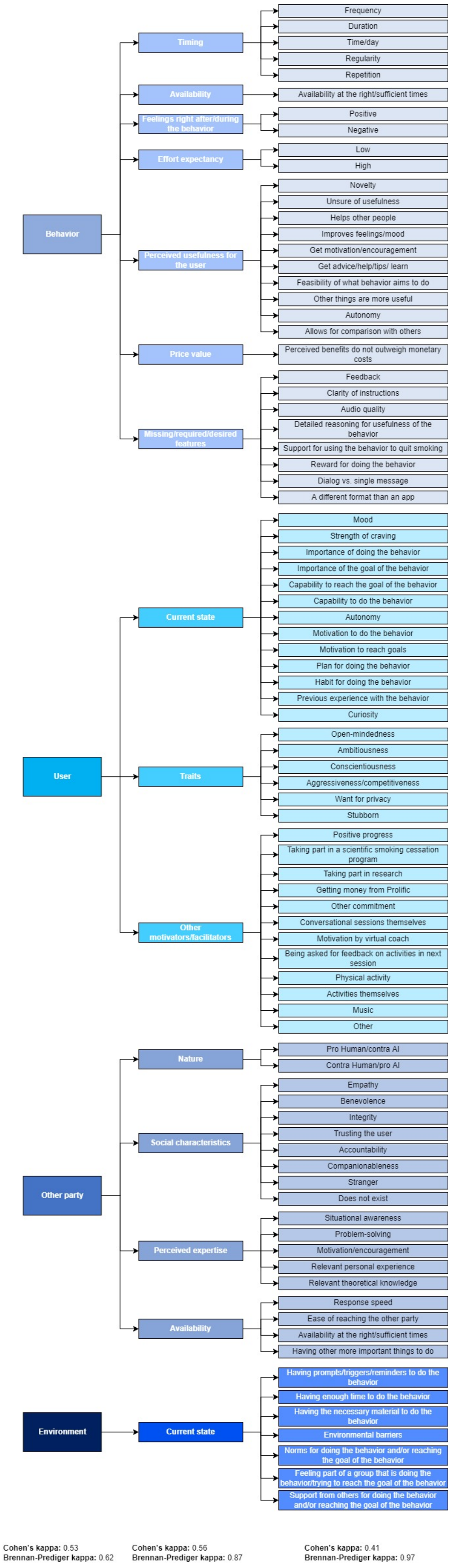

Figure S7: **Coding scheme.** The coding scheme consists of 4 codes at the highest level, 15 codes at the second level, and 86 codes at the third level. We show the reliability for each coding level.
