# Supplementary material for: Users’ needs for a digital smoking cessation application and how to address them: A mixed-methods study
Source: PeerJ. 2022 Aug 19;10:e13824. doi: 10.7717/peerj.13824 (PMC9394512; doi:10.7717/peerj.13824)
Supplement: Supplemental Information 8 — The example shows the start of the second conversational session with Sam, including responses given by a participant. [file peerj-10-13824-s008.pdf]

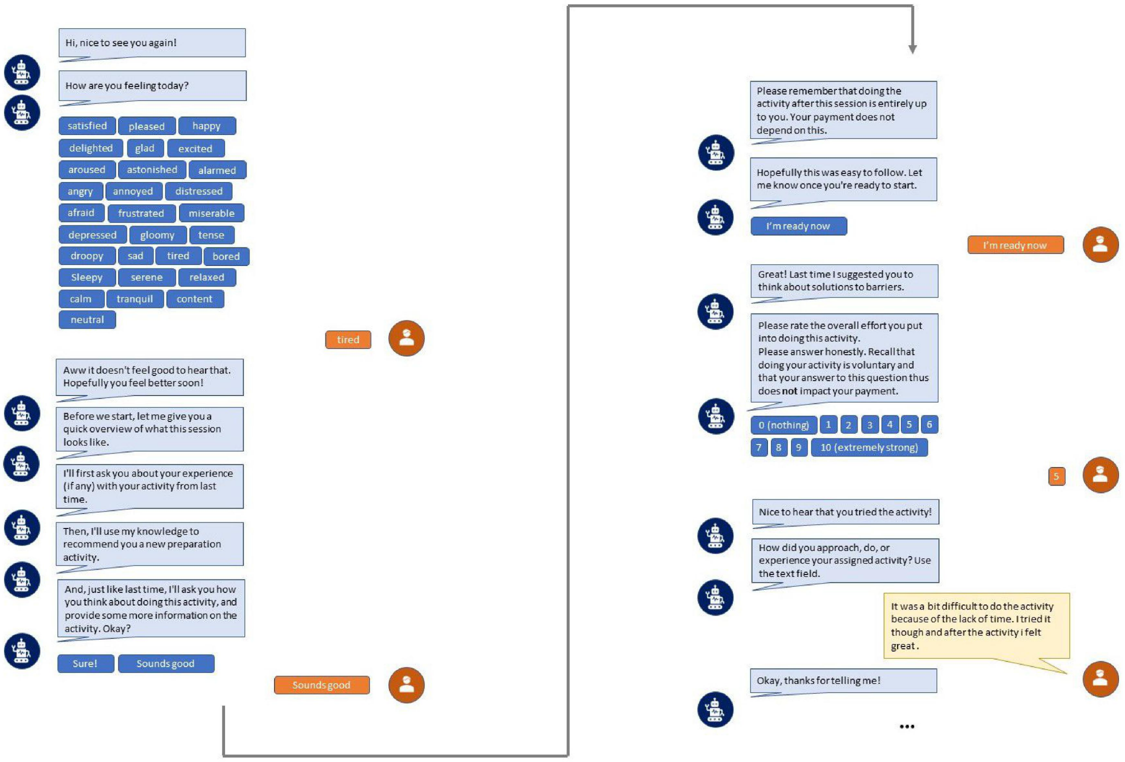

Figure S8: **Conversation example.** The example shows the start of the second conversational session with Sam, including responses given by a participant.
