# Supplementary material for: Users’ needs for a digital smoking cessation application and how to address them: A mixed-methods study
Source: PeerJ. 2022 Aug 19;10:e13824. doi: 10.7717/peerj.13824 (PMC9394512; doi:10.7717/peerj.13824)
Supplement: Supplemental Information 9 — The response types are the activity experiences, barriers, motivators, and the groups of interaction scenarios. N denotes the number of responses. [file peerj-10-13824-s009.pdf]

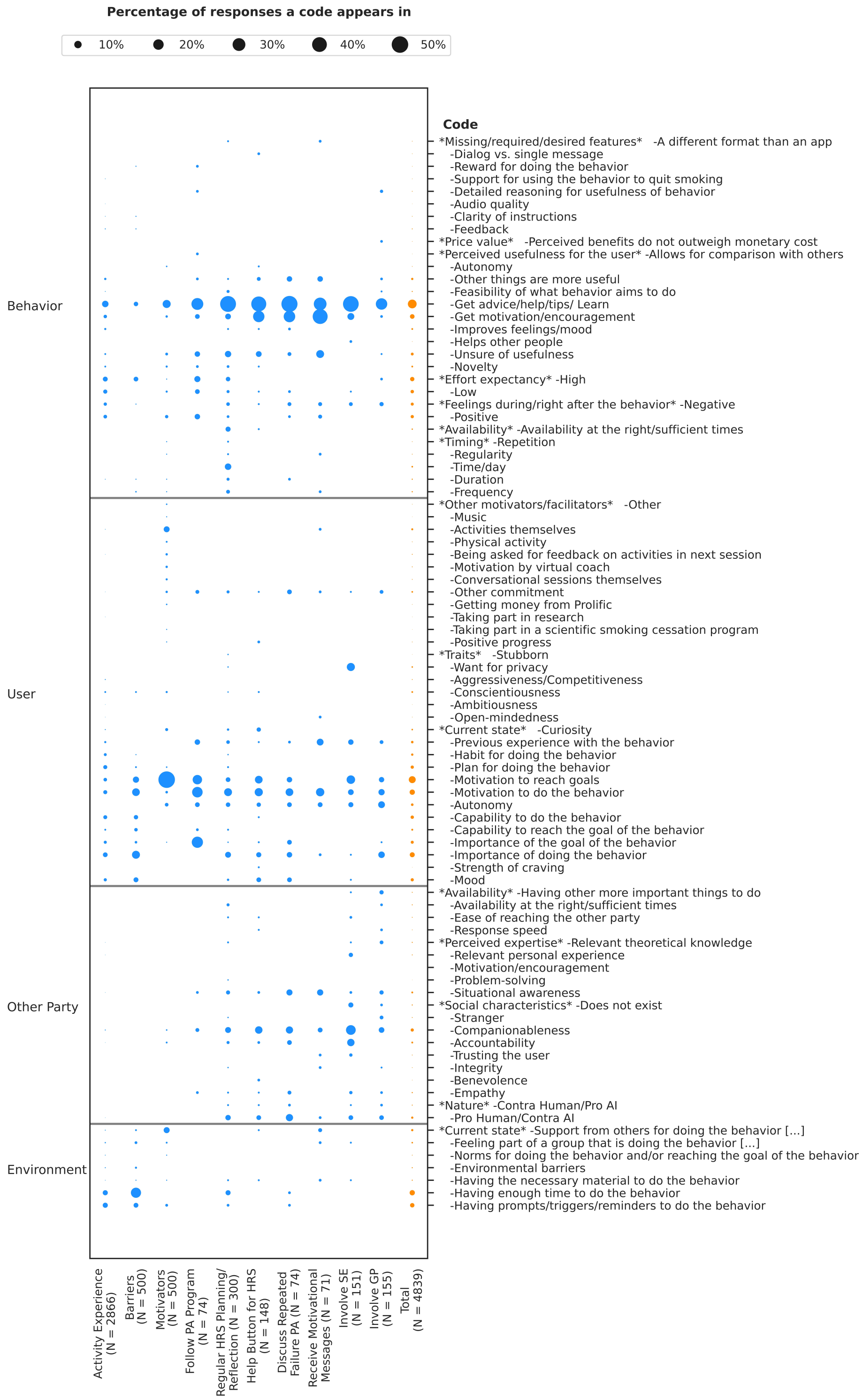

Figure S9: **Percentage of times each code from the coding scheme appears in each response type as well as across all response types together.** The response types are the activity experiences, barriers, motivators, and the groups of interaction scenarios. N denotes the number of responses.
